# Effects of Melt Temperature and Non-Isothermal Flow in Design of Coat Hanger Dies Based on Flow Network of Non-Newtonian Fluids

**DOI:** 10.3390/polym14153161

**Published:** 2022-08-03

**Authors:** Amin Razeghiyadaki, Dongming Wei, Asma Perveen, Dichuan Zhang, Yanwei Wang

**Affiliations:** 1 Department of Mathematics, School of Sciences and Humanities, Nazarbayev University, Nur-Sultan 010000, Kazakhstan; amin.razeghiyadaki@nu.edu.kz; 2 Department of Mechanical & Aerospace Engineering, School of Engineering and Digital Sciences, Nazarbayev University, Nur-Sultan 010000, Kazakhstan; asma.perveen@nu.edu.kz; 3 Department of Civil & Environmental Engineering, School of Engineering and Digital Sciences, Nazarbayev University, Nur-Sultan 010000, Kazakhstan; dichuan.zhang@nu.edu.kz; 4 Department of Chemical & Materials Engineering, School of Engineering and Digital Sciences, Nazarbayev University, Nur-Sultan 010000, Kazakhstan; yanwei.wang@nu.edu.kz; 5 Laboratory of Computational Materials Science, Center for Energy and Advanced Materials Science, National Laboratory Astana, Nur-Sultan 010000, Kazakhstan

**Keywords:** polymer processing, sheet die design, coat-hanger die, non-isothermal non-Newtonian fluids, constant shear-rate die, viscous dissipation, temperature effects

## Abstract

In the design of coat hanger extrusion dies, the main objective is to provide a uniform flow rate at the die exit. Previously, a multi-rheology isothermal method model for coat hanger extrusion dies was developed to reach this objective. Polymer melts in extrusion dies commonly experience high shear rates. Viscous dissipation rooted by high shear rate may lead to significant temperature differences across the die. Due to temperature-dependency of viscosity, temperature differences may lead to nonuniform flow rates, which may significantly affect the flow rate at the die exit. As a result, a new design method is proposed to take into account the effects of temperature and viscous dissipation in the design of coat hanger dies. Although more non-Newtonian fluid rheology models can be adapted in the proposed study, as demonstration, temperature-dependent power-law and Carreau–Yasuda models are adapted in this study. Performances are compared with our isothermal method published earlier. In addition, the novel nonisothermal method is comprehensively examined where the effect of viscous dissipation is studied through Brinkman number of extrusion die. It is demonstrated that, for a low Brinkman number, both isothermal and nonisothermal design give similar flow uniformity level. However, for higher Brinkman numbers, the proposed nonisothermal method produces a design with more desirable velocity uniformity level along with a maximum improvement of 5.24% over the isothermal method. In addition, dependency of flow field on temperature, due to temperature-dependent viscosity, is studied, and it is demonstrated that fully-developed velocity profile changes as temperature increases along the flow channel. Moreover, the effect of the temperature sensitivity parameter in temperature-dependent non-Newtonian models is considered. It is demonstrated that the temperature boundary condition with the Biot number of 1.0 gives adequate results for lower values of the temperature sensitivity parameter.

## 1. Introduction

The most challenging aspect of extrusion die design is providing a uniform exit velocity profile, hence no or reduced correction is required by adjusting bolts. Nonlinear behavior of viscosity of polymer melts and complicated multi-physics of extrusion dies make extrusion die design a challenging task. In addition, temperature rise due to viscous dissipation and temperature dependency of viscosity adds more complexity to the flow rate profile at die exit where a uniform profile is needed. Since the internal geometry of die and the process conditions determine optimal performance of extrusion dies, numerical methods are required to carry out inter-dependency of fluid flow and its dependency on temperature and nonlinear viscosity. Generally, a trial and error approach is adapted to test different designs to reach to an optimal design [1,2]. This approach requires a very high number of costly simulations to be carried out.

High computational cost of numerical methods limits numerical design optimization in engineering applications. Thus, introduction of simplified methods with low computational cost with efficiency is necessary. Different design methods based on analytical [3,4,5], semi-analytical [6,7] and numerical optimization were developed [8]. Winter and Fritz [3] proposed the first analytical design method for rectangular and circular-shaped sheeting extrusion dies. Degradation appears to be the main issue in extrusion of temperature-sensitive polymers. Awe et al. [4] proposed a shortened Winter–Fritz model to avoid this issue.

Semi-analytical methods based on a flow network method (FNM) are also reported in the literature. A flow network analysis, also known as a hydraulic-electric circuit analogy, uses the conventional concept of electric circuit theory for an analysis of fluid flow problems [9]. Some literature demonstrates [7,9] the application of this method for coat-hanger die design with different rheology models. Michaeli et al. [1] combined the finite element method with the network theory to find the optimum velocity distribution. Optimized designs for rectangular and circular shaped manifolds are achieved using power-law and Carreau–Yasuda rheology models. In addition, Yilmaz and Kadikkopru [7] developed a model based on FNM which successfully achieved optimal geometrical parameters in the manifold leading to uniform velocity profiles. Using a trial and error approach, Igali et al. [10] optimized flow distribution of sheeting extrusion die with finite element simulations. The above researchers did not consider temperature effects in their designs.

Due to temperature dependency of viscosity, temperature field can change flow field. As a result, the effect of temperature on flow uniformity was studied in literature. Lebaal et al. [11] optimized wall temperature and flow rate of a wire-coating die with Kriging interpolation and a sequential quadratic programming algorithm optimization method. Their study concluded with the possibility to design a coat-hanger wire-coating die for a different polymer range and flow rates. In another study, Lebaal et al. [12] studied the effectiveness of the response surface method and Sequential Quadratic Programming for optimization of wall temperature to gain the best velocity profile at die exit. Wu et al. [13] also developed a one-dimensional model that takes into account the effect of temperature variation and its effect on flow uniformity. In another study, Lebaal et al. [14] optimized the wall temperature profile of a coat-hanger extrusion die. By changing wall temperature, viscosity of the fluid in the vicinity of the wall can be changed and consequently flow rate can be manipulated. In a further study, Lebaal [12] presents a Kriging Swarm Optimization (KSO) algorithm for optimization of three geometrical design parameters to achieve a uniform velocity profile of a coat hanger die.

To the authors’ best knowledge, no analytical or semi-analytical design method of a coat hanger die which takes into account the effects of temperature exists in literature. In our previous study, a new isothermal design method based on constant shear rate and uniform velocity assumptions was proposed [6]. It is known that the design of a coat hanger dies depends on wall shear rate, temperature and heat dissipation of polymer melt [15]. Therefore, a previous model is modified to take into account the effects of temperature and heat dissipation, and its subsequent effect on apparent viscosity of the polymer. This newly proposed model takes into account different non-Newtonian models. Based on our proposed model, new design curves are produced, and their performance is discussed through pressure, temperature and shear rate distribution.

## 2. Model and Methods

**Figure 1:**
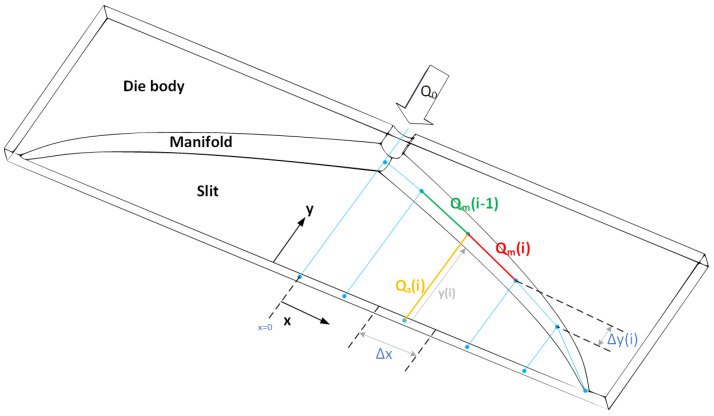
Schematic of an extrusion die.

A sheeting extrusion die is made of two parts, the slit and the manifold, as shown in Figure 1. Due to symmetry, only half of the die is considered here. Since a uniform flow is desired at the exit of the die, the manifold has to be designed in such a manner that satisfies this goal. Manifold can be characterized by two geometrical parameters, i.e., distance from center of manifold to die exit y(x) and radius R(x) of the manifold. Flow rates in each manifold and slit segment are denoted by $Q_{m}\left(i\right)$ and $Q_{s}\left(i\right)$, respectively. Vertical and horizontal distance between two adjacent manifold segments are denoted by Δy and Δx, respectively. Length between to adjacent manifold nodes is denoted by Δζ and is equal to $\sqrt{{\Delta x}^{2}+{\Delta y}^{2}}$.

### 2.1. Flow Network Method for Die Design

In our previous work [6], we proposed simplifying the die into a series of segments where fluid flows in each segment can be described analytically or numerically. As a result of segmentation, two continuous geometrical parameters reduce to two sets of discrete parameters. Two sets of equations are needed to be solved simultaneously to find these two geometrical parameters of extrusion. The following assumptions are made for the model proposed in this study:

- Steady-state, non-isothermal, incompressible flow;
- Streamlined flow;
- Uniform pressure and flow rates at die exit;
- Unidirectional and fully-developed flow in both manifold and slit;
- Constant wall shear rate in the manifold and the slit.

The constant wall shear rate assumption results in the following equation:

(1) $${\dot{\gamma }}_{m}\left(R\right)={\dot{\gamma }}_{s}\left(y\right)$$

where $\dot{\gamma }$ is shear rate. Subscripts m and s refer to manifold and slit. Constant wall shear rate also results in the following equation known as the Winter–Fritz [3] equation:

(2) $$\frac{dy}{dx}=-\;{\left[{\left(\frac{{\left(dp/dy\right)}_{s}}{{\left(dp/d\zeta \right)}_{m}}\right)}^{2}-1\right]}^{-1/2};$$

in discretized form, Equation (2) can be written as follows:

(3) $$\left(y_{i+1}-y_{i}\right)=-\left(x_{i+1}-x_{i}\right){\left[{\left(\frac{{\left(\frac{dp}{dy}\right)}_{s}\left(i\right)}{{\left(\frac{dp}{d\zeta }\right)}_{m}\left(i\right)}\right)}^{2}-1\right]}^{-1/2}$$

(4) $$\left(y_{i+1}-y_{i}\right)=-\left(x_{i+1}-x_{i}\right){\left[{\left(\frac{\frac{\Delta p_{s,i}}{L_{s,i}}}{\frac{\Delta p_{m,i}}{L_{m,i}}}\right)}^{2}-1\right]}^{-1/2}$$

where $\Delta p_{s,i}$, $\Delta p_{m,i}$, $L_{s,i}$ and $L_{m,i}$ are pressure drops and corresponding length of the slit and the manifold, respectively. Since no analytical simple equation is available for pressure gradient of nonisothermal non-Newtonian fluid flows, a numerical method is adapted. Since uniform flow rates is assumed at die exit:

(5) $$Q_{s}\left(i\right)=Q_{s}=\frac{Q_{0}}{N+1}\;\;\mathrm{where}\;1\leq i\leq N$$

where *N* and Q are the total number of segments and flow rate. Mass conservation for each node on manifold gives the following:

(6) $$Q_{m}\left(i-1\right)=Q_{m}\left(i\right)+Q_{s}\left(i\right)\;\;\mathrm{where}\;\;1\leq i\leq N$$

and

(7) $$Q_{m}\left(N\right)=Q_{s}\;\;Q_{m}\left(0\right)=Q_{0}$$

from Equations (5) and (6); flow rates in both manifold and slit are calculated.

**Figure 2:**
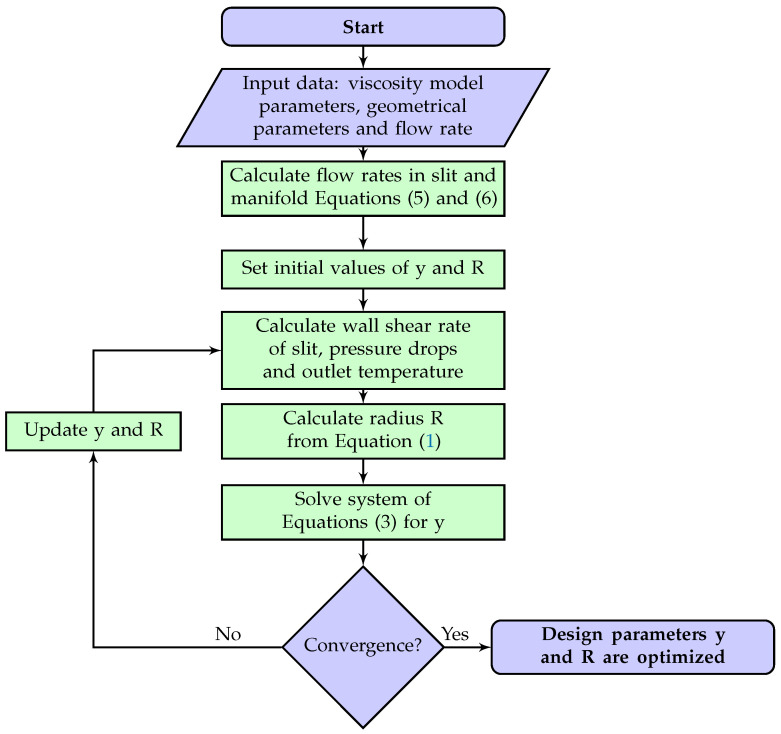
Algorithm used in the proposed flow network method for die design.

The algorithm of calculations is shown in Figure 2. The first step is initialization of parameters and calculation of flow rates in each segment. Next, shear rates, pressure drops and outlet temperature are calculated. With calculated shear rate, the radius of manifold is calculated by Equation (4). Lastly, with calculated pressure drops, new *y*-values are calculated by Equation (4). Calculations are repeated until convergence is reached. The convergence criterion is set to $10^{-5}$. The convergence criterion is defined as follows:

(8) $$max\{|\frac{y{\left(i\right)}^{\mathrm{old}}-y{\left(i\right)}^{\mathrm{new}}}{y{\left(i\right)}^{\mathrm{old}}}|,|\frac{R{\left(i\right)}^{\mathrm{old}}-R{\left(i\right)}^{\mathrm{new}}}{R{\left(i\right)}^{\mathrm{old}}}|\}<\varepsilon \;\;1<i<N$$

Computational times were between 20 to 120 min on a core i9 personal computer.

### 2.2. Non-Newtonian Models

For demonstration, temperature-dependent power-law and Carreau–Yasuda rheology models are considered. The shear-rate and temperature-dependent viscosity function of the Power-law model is as follows:

(9) $$\eta \left(\dot{\gamma },T\right)=m\left(T\right){\dot{\gamma }}^{n-1}$$

where m(T) is often referred to as the flow consistency index and *n* as the flow behavior index. The temperature dependence of the flow consistency index is given by

(10) $$m\left(T\right)=m_{0}\exp\left[-\alpha_{pl}\left(T-T_{\mathrm{ref}}\right)\right]$$

where $\alpha_{pl}$ is the temperature sensitivity parameter.

The Carreau–Yasuda model with temperature dependence is given as follows [16]:

(11) $$\eta \left(\dot{\gamma },T\right)/a_{T}=\eta_{\infty }+\left(\eta_{0}-\eta_{\infty }\right){\left[1+{\left(a_{T}\dot{\gamma }\lambda \right)}^{a}\right]}^{(n-1)/a}$$

where $a_{T}=\exp\left[-\alpha_{cs}\left(T-T_{\mathrm{ref}}\right)\right]$ is often referred to as the shift factor; $\alpha_{cs}$ corresponds to the temperature sensitivity parameter.

### 2.3. Pressure Drop Calculation

In each segment of the manifold, the flow is assumed to be unidirectional. In addition, fluid flow is assumed to be laminar, hydrodynamically fully developed and thermally developing in channel segment direction. The non-Newtonian fluid enters at temperature $T_{0}$, and the effect of viscous dissipation is considered here. Flow in a slit segment can be assumed as a planar flow between two parallel plates and flow in a manifold segment can be assumed as pipe flow for a circular manifold, respectively. Polymer melts have high Prandtl number $\mathrm{Pr}=c_{p}\mu /k_{t}$, where $k_{t}$, μ, and $c_{p}$ are the thermal conductivity, apparent viscosity and heat capacity, respectively; and therefore velocity profile can be assumed to be fully-developed [17,18,19,20,21,22]. Due to coupling between velocity and temperature fields, energy and momentum equations are needed to be solved simultaneously. By solving the following equations, velocity and temperature profiles can be calculated for planar (κ=0) and pipe flows (κ=1):

(12) $$\begin{array}{cc} \; & \mathrm{Momentum}\;\mathrm{balance}:\;-\frac{dp}{dx_{2}}+\frac{d\tau }{dx_{1}}=0\\ \; & B.C.1:\;\frac{\partial u}{\partial x_{1}}=0\;\mathrm{at}\;x_{1}=0\\ \; & B.C.2:\;u=0\;\mathrm{at}\;x_{1}=L_{c} \end{array}$$

(13) $$\begin{array}{cc} \; & \mathrm{Energy}\;\mathrm{balance}:\;\;\rho c_{p}u\frac{\partial T}{\partial x_{2}}=\frac{k_{t}}{x_{1}^{\kappa }}\frac{\partial }{\partial x_{1}}\left(x_{1}^{\kappa }\frac{\partial T}{\partial x_{1}}\right)+\eta {\left(\frac{du}{dx_{1}}\right)}^{2}\\ \; & B.C.1:\;T=T_{0}\;\mathrm{at}\;x_{2}=0\\ \; & B.C.2:\;\frac{\partial T}{\partial x_{1}}=0\;\mathrm{at}\;x_{1}=0\\ \; & B.C.3:\;-k_{t}\frac{\partial T}{\partial x_{1}}=h\left(T-T_{R}\right)\;\mathrm{at}\;x_{1}=L_{c} \end{array}$$

where *u*, *T*, *p*, τ, $x_{1}$, $x_{2}$ and $L_{c}$ are velocity, temperature, pressure, shear stress and transverse, axial coordinates and characteristic length (radius of manifold segment or height of slit segment), respectively. It is worth notiing that both Equations (12) and (13) are valid for both planar flow and pipe flows. In case of pipe flow, $x_{1}$ is the radius, while, for the case of planar flow, $x_{1}$ is the distance from the center plane between the two parallel plates. In other words, $x_{1}=0$ is axis of pipe or mid plane between the two parallel plates. Due to symmetry, boundary conditions at $x_{1}$ for both momentum and energy balance equations are set to zero gradient. Physical properties such as viscosity, density, constant pressure heat capacity and thermal diffusivity are denoted by μ, ρ, $c_{p}$ and $\alpha_{T}$, respectively. Apparent viscosity μ is a function of both shear rate and temperature. Shear stress and thermal diffusivity by definition are given as follows:

(14) $$\tau =\eta \left(\dot{\gamma },T\right)\dot{\gamma }\;\;\alpha_{T}=\frac{k_{t}}{\rho c_{p}}$$

By solving these Equations (12) and (13), temperature, pressure drop and wall shear rate (velocity gradient at wall) can be calculated.

**Figure 3:**
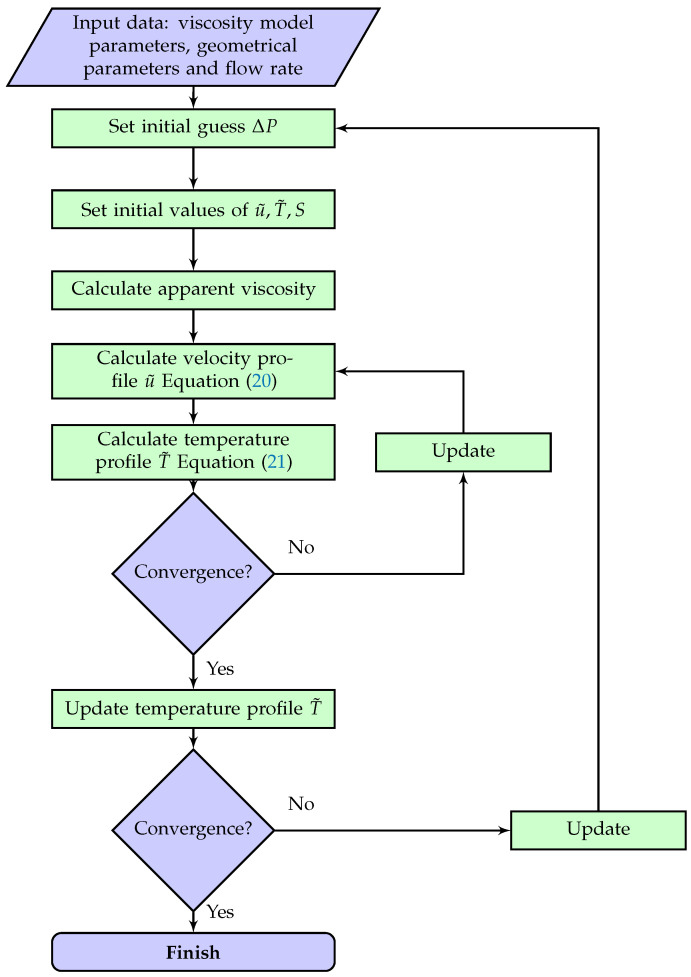
Algorithm of calculation of pressure drop.

By non-dimensionalization of the momentum and the energy equations (Equations (12) and (13)) by the parameters in Table 1, we have for the momentum balance equation:

(15) $$S=\frac{d\tilde{u}}{d{\tilde{x}}_{1}}=\frac{{\tilde{x}}_{1}}{\tilde{\eta }}$$

and for the energy balance equation:

(16) $$\begin{array}{cc} \; & \tilde{u}\frac{\partial \tilde{T}}{\partial {\tilde{x}}_{2}}=\frac{1}{{\tilde{x}}_{1}^{\kappa }}\frac{\partial }{\partial {\tilde{x}}_{1}}\left({\tilde{x}}_{1}^{\kappa }\frac{\partial \tilde{T}}{\partial {\tilde{x}}_{1}}\right)+\mathrm{Br}\;S{\tilde{x}}_{1}\\ \; & B.C.1:\;\tilde{T}=1\;at\;{\tilde{x}}_{2}=0\\ \; & B.C.2:\;\frac{\partial \tilde{T}}{\partial {\tilde{x}}_{1}}=0\;at\;{\tilde{x}}_{1}=0\\ \; & B.C.3:\;-\frac{\partial \tilde{T}}{\partial {\tilde{x}}_{1}}=\mathrm{Bi}\;\tilde{T}\;at\;{\tilde{x}}_{1}=L_{c} \end{array}$$

the Brinkman number, and the Biot number are defined as:

(17) $$\begin{array}{ccc} \mathrm{Br} & = & \frac{\tau_{w}{\dot{\gamma }}_{w}L_{c}^{2}}{k_{t}\left(T_{0}-T_{R}\right)} \end{array}$$

(18) $$\begin{array}{ccc} \mathrm{Bi} & = & \frac{h_{ext}L_{c}}{k} \end{array}$$

the non-dimensional viscosity $\tilde{\eta }\left(\dot{\gamma },T\right)$ for the power-law model (Equation (9)) and the Carreau–Yasuda model (Equation (11)) are defined in Table 2 and Table 3, respectively, where shear rate at wall is calculated as follows:

(19) $$\begin{array}{cc} \; & \eta \left({\dot{\gamma }}_{w},T_{w}\right){\dot{\gamma }}_{w}=\frac{\Delta p}{2LR}\;\mathrm{for}\kappa =1(\mathrm{manifold}\mathrm{segment}\mathrm{flow})\\ \; & \eta \left({\dot{\gamma }}_{w},T_{w}\right)\dot{\gamma_{w}}=\frac{\Delta p}{2LH}\;\mathrm{for}\kappa =0(\mathrm{slit}\mathrm{segment}\mathrm{flow}) \end{array}$$

the characteristic length $L_{c}$ depends on the cross-section (radius $r_{0}$ for circular and half distance of plates H/2 for planar channel flow). Arpin et al. [23] suggested a Biot number of 1.0 as the temperature boundary condition of coat hanger dies. Our computational fluid dynamics (CFD) simulation confirmed this assumption. The algorithm for calculation of pressure drop is shown in Figure 3. Discretization results are as follows:

(20) $$\frac{\tilde{u}\left(i+1\right)-\tilde{u}\left(i\right)}{{\tilde{x}}_{1}\left(i+1\right)-{\tilde{x}}_{1}\left(i\right)}=\frac{{\tilde{x}}_{1}\left(i\right)}{\tilde{\eta }\left(i\right)}$$

(21) $$\begin{array}{cc} \left[\frac{\tilde{u}\left(i\right)}{{\Delta \tilde{x}}_{2}}+\frac{2}{{\left(\Delta {\tilde{x}}_{1}\right)}^{2}}\right]\tilde{T}\left(i,j\right) & +\left[\frac{2}{{\left(\Delta {\tilde{x}}_{1}\right)}^{2}}+\frac{\kappa }{{\tilde{x}}_{1}\left(i\right)\Delta {\tilde{x}}_{1}}\right]\tilde{T}\left(i-1,j\right)\\ & +\left[\frac{2}{{\left(\Delta {\tilde{x}}_{1}\right)}^{2}}+\frac{-\kappa }{{\tilde{x}}_{1}\left(i\right)\Delta {\tilde{x}}_{1}}\right]\tilde{T}\left(i+1,j\right)=\frac{\tilde{u}\left(i\right)}{{\Delta \tilde{x}}_{2}}\tilde{T}\left(i,j-1\right)+\mathrm{Br}S_{i}^{2} \end{array}$$

### 2.4. Verification and Validation by CFD

**Figure 4:**
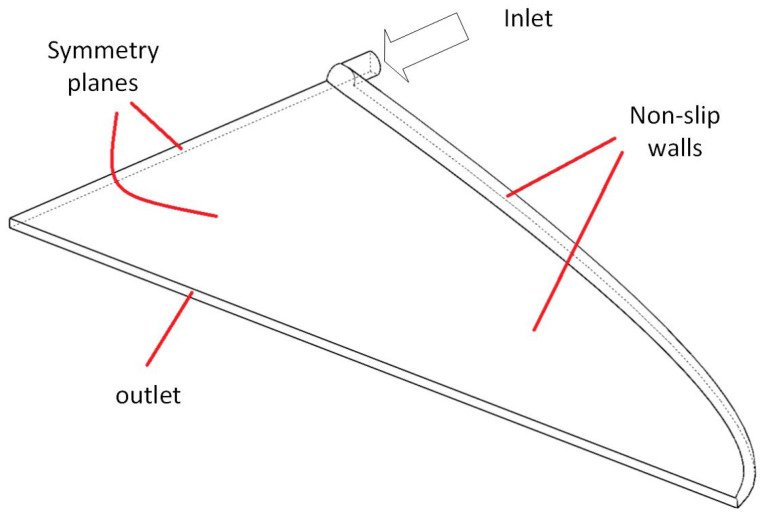
Computational domain and boundary conditions for CFD analysis.

CFD simulation is used as a tool to compare the results of the proposed model and those of the previous method [6]. The Ansys Fluent 2021.R2 finite volume CFD software package is adapted for this purpose. Computation domain is shown in Figure 4. Due to symmetry, only a quarter of coat-hanger dies are considered as the computation domain. The continuity, momentum and energy equations must be considered and solved simultaneously:

(22) ∇·U=0

(23) ρU∇·U=−∇p+∇·τ

(24) $$\rho c_{p}\left(U\cdot \nabla \right)T=\nabla \left(k\nabla T\right)+\tau :\nabla U$$

At the inlet of the computation domain, uniform velocity and temperature profile are applied. At the die exit, zero gauge pressure is assumed. Non-slip and Biot = 1 (Equation (13)) boundary conditions are applied to solid walls. A C++ user defined function is written to take into account viscous dissipation as a source term in the energy equation. For accurate representation of 3D geometry, a Python code in Ansys Spaceclaim 19.1 is written.

## 3. Results and Discussion

### 3.1. Design Curves

**Figure 5:**
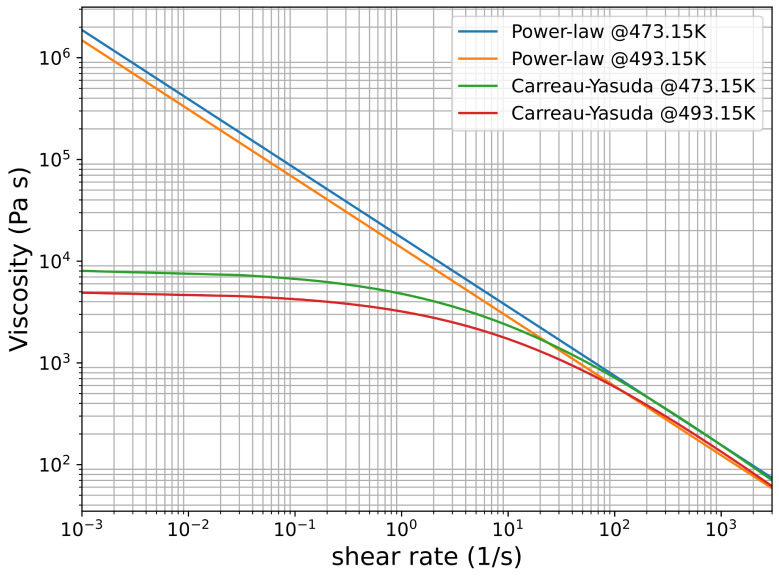
Viscosity versus shear rate of Power-law and Carreau–Yasuda at 473.15 K and 493.15 K, respectively.

In this work, values of the power-law model parameters are $m_{0}$ = 17,092 $\;\mathrm{Pa}\;\cdot \;s^{n}$, n=0.32, $\alpha_{pl}=0.011624\;K^{-1}$, and $T_{\mathrm{ref}}=473.15\;K$; The Carreau–Yasuda model parameters are $\eta_{0}=8234\;\mathrm{Pa}\;\cdot \;s$, $\eta_{\infty }=0\;\mathrm{Pa}\;\cdot \;s$, λ=0.129s, n=0.217, a=0.468, $\alpha_{cs}=0.025\;K^{-1}$ and $T_{\mathrm{ref}}=473.15\;K$. Figure 5 shows viscosity against shear rate of rheology models for both power-law and Carreau–Yasuda models.

**Figure 6:**
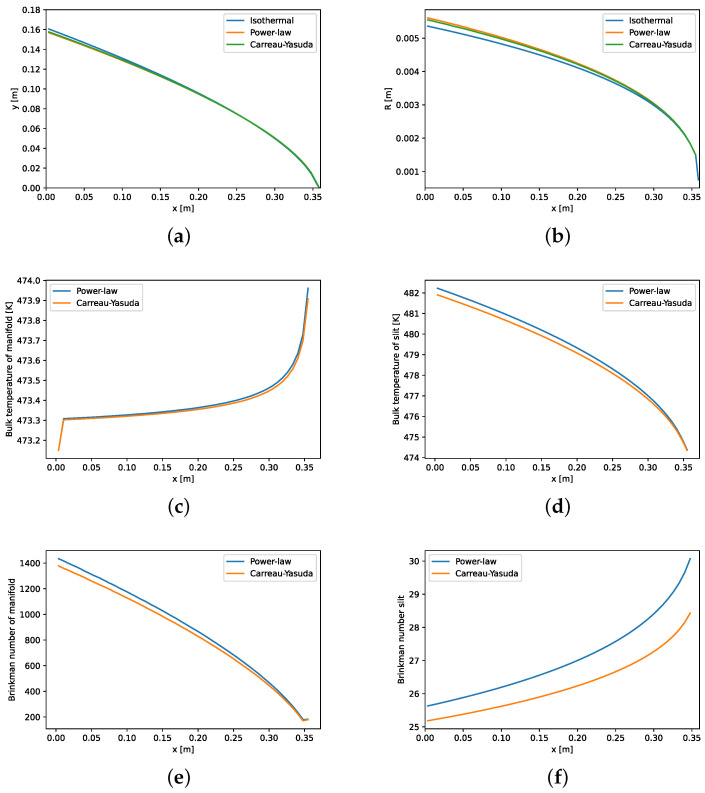
As a result of isothermal and isothermal designs: (**a**) die land length; (**b**) manifold radius; bulk temperatures of (**c**) manifold; and (**d**) slit and Brinkman number of (**e**) manifold and (**f**) slit for temperature-dependent power-law and Carreau–Yasuda fluid. See Table 4 for input parameters.

Figure 6 depicts nonisothermal design curves for the power-law and the Carreau–Yasuda fluids. Input parameters for design and optimization are shown in Table 4. The isothermal power-law designs [6] for the otherwise same given parameters are also shown in Figure 6 as a comparison. Due to temperature changes across the die, calculated wall shear rate is non-uniform. This results in different radii as given by Equation (1). Design curves of *y* and radius for both power-law and Carreau–Yasuda fluids are almost the same. Calculated wall shear rates are in the range of 500 to 600 s ${}^{-1}$. In this range, both power-law and Carreau–Yasuda fluids behave in a similar manner as shown in Figure 5. Fluid flows through the manifold, it accumulates more energy from viscous dissipation and bulk temperature in manifold increases as shown in Figure 6c. The die has longer die land length at the center than its edges (Figure 1), thus fluid exits the slit at a higher temperature at the center than the edges as shown in Figure 6d–f, which depicts Brinkman numbers in the manifold and the slit, respectively. Flow rate decreases in the *x*-direction in the manifold, resulting in a decrease in the shear rate and subsequently Brinkman number in the manifold. As shown in Figure 6d, the slit temperature is deceasing from the center to the edge of the die, which results in high viscosity and higher Brinkman number, as shown in Figure 6f. For the given process conditions, the temperature across the die exit changes approximately 8 K as shown in in this figure.

### 3.2. Effect of Heat Viscous Dissipation

Effect of viscous dissipation can be quantitatively studied by the Brinkman number, which is defined as ratio of heat generation due to heat dissipation to heat transfer to wall. Since viscous dissipation across the die is changing, a new overall Brinkman number is defined based on Brinkman number of power-law fluid in the literature [24]. The overall Brinkman number of die for a power-law fluid is defined as follows:

(25) $${\mathrm{Br}}_{\mathrm{die}}=\frac{m_{0}b^{2}{Q_{0}}^{n+1}}{k_{t}\left(T_{0}-T_{R}\right){\left(b^{2}h_{s}\right)}^{n+1}.}$$

Four cases with different Br are defined as shown in Table 5. The only parameter that changes is the power-law consistency factor $m_{0}$. Values for consistency factors are $10^{2}$, $5\times 10^{3}$, $1\times 10^{4}$ and $1.5\times 10^{4}$, which corresponds to Brinkman numbers of 2.42, 122, 243 and 365.

**Figure 7:**
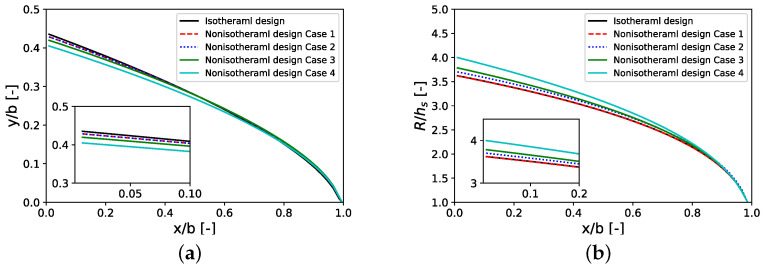
Nonisothermal designs for different cases (**a**) die land length *y*; (**b**) manifold radius *R*; see Table 5 and Table 6 for the parameters used.

Figure 7 depicts the designs for all cases. It is worth mentioning that, for the isothermal method, all cases give the same design curves, since the isothermal method is independent of consistency factor. On the other hand, the nonisothermal method is affected by viscous dissipation, which is dependent on Brinkman number and subsequently on consistency factor, as shown in Equation (25). As shown in Figure 7, with an increase in Brinkman number, the radius of manifold increases while die land length y decreases. An increase in manifold radius leads to a lower shear rate and therefore lower heat generation due to viscous dissipation. Additionally, a lower die land length corresponds to a shorter travel length of polymer through a slit and hence a lower effect of heat viscous dissipation.

Table 6 also shows the velocity uniformity index of all cases based on CFD simulations. The uniformity level of velocity distribution is measured and defined by uniformity index as follows:

(26) $$\phi =\frac{\sum_{i}^{N}\left|v_{i}-v_{\mathrm{ave}}\right|dA_{i}}{Av_{\mathrm{ave}}}$$

**Figure 8:**
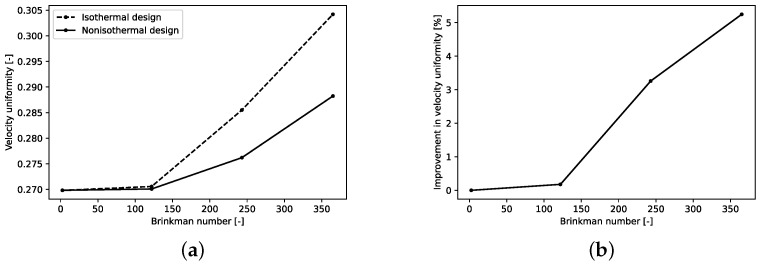
Nonisothermal design performance and its comparison to previous design (**a**) isothermal and nonisothermal design velocity uniformity index and (**b**) percentage of improvement of uniformity of velocity profile; see Table 5 and Table 6 for the parameters used.

**Figure 9:**
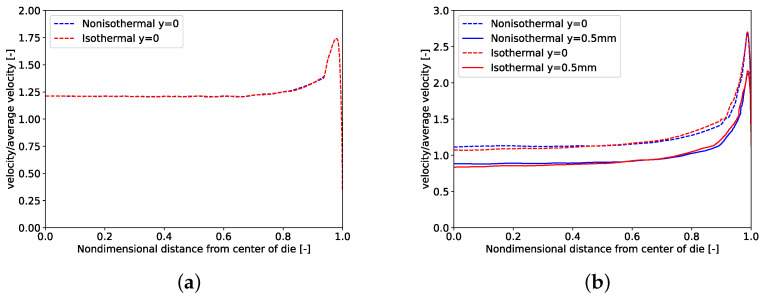
Comparison of isothermal and nonisothermal designs velocity profiles (CFD) for (**a**) Case 1 with ${\mathrm{Br}}_{\mathrm{die}}=2.42$ and (**b**) Case 4 with ${\mathrm{Br}}_{\mathrm{die}}=365$. Dashed lines correspond to the center line at the exit, and solid lines correspond to the line half-way between the center and the slit wall.

Figure 8 depicts improvement in velocity uniformity index of the nonisothermal design over the isothermal one. As shown in Figure 8a, with an increase in Brinkman number, more improvement in ϕ can be seen. This is due to the effect of viscous dissipation on temperature and subsequently on hydrodynamics of the extrusion die. For low Brinkman numbers, isothermal and nonisothermal designs result in similar die geometry and similar velocity distribution at the exit of the die. However, with an increase in viscous dissipation, improvement in the velocity uniformity index can be seen in almost linear fashion (Figure 8b). Thus, for fluids with temperature dependent viscosity, previous design methods are not sufficient and effects of viscous dissipation are necessary to be considered. Nonisothermal design for case 4 with Brinkman number 365 shows 5.24% improvement in velocity uniformity index compared to the isothermal design. Figure 9 shows isothermal and nonisothermal velocity profiles at the center line of the die exit for Brinkman numbers of 2.42 and 365. Both velocity profiles of nonisothermal and isothermal cases for both Brinkman numbers behave; similarly, there is a flat and uniform profile everywhere except a peak at the edge of the die where the manifold reaches the die exit. As shown in this figure, the value of peak increases as Brinkman number increases.

### 3.3. Effect of Temperature on Pressure Drop vs. Flow Rate Relation

**Figure 10:**
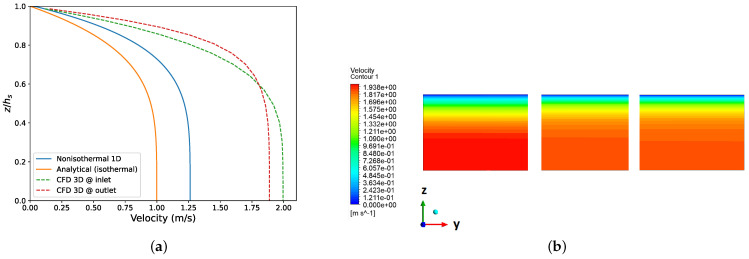
Velocity distribution of a power law fluid in a slit segment: (**a**) velocity profiles obtained through isothermal analytical, one-dimensional, numerical and three-dimensional numerical (CFD) methods; (**b**) contour plot of velocity (CFD) at three different sections of channels. See Table 7 for values of the parameters used.

Any inaccuracy in calculation of pressure drops in Equation (4) results in inaccurate design curve (y). For simplicity, in this study, the velocity profile is assumed to be fully-developed while the temperature profile varies in the axial direction of a slit or manifold segment. Due to dependency of viscosity on temperature, the actual velocity profile is changing along the axial direction. Figure 10a shows a comparison of analytical (isothermal), one-dimensional, numerical (Equations (15) and (16)) and three-dimensional numerical (CFD) velocity profiles for a power-law fluid at one segment of the slit. Input parameters for all cases are given in Table 7. Due to dependency of viscosity on temperature, higher velocities are obtained by both numerical nonisothermal calculations. In opposition to the one-dimensional calculation, CFD simulation gives different velocity profiles at inlet and outlet of slit regions, as shown in Figure 10b. Despite the fact that the one-dimensional, numerical method is not an accurate representation of three-dimensional profiles, it provides better estimation than the isothermal method.

### 3.4. Effect of Temperature Sensitivity Parameter

In the temperature-dependent viscosity model, the intensity of temperature dependency is determined by the temperature sensitivity parameter ($\alpha_{pl}$ or $\alpha_{cs}$). The higher value of the temperature sensitivity parameter leads to higher dependency of viscosity on temperature. Therefore, it is paramount to study how the temperature sensitivity parameter affects design and subsequently flow distribution at die exit.

**Figure 11:**
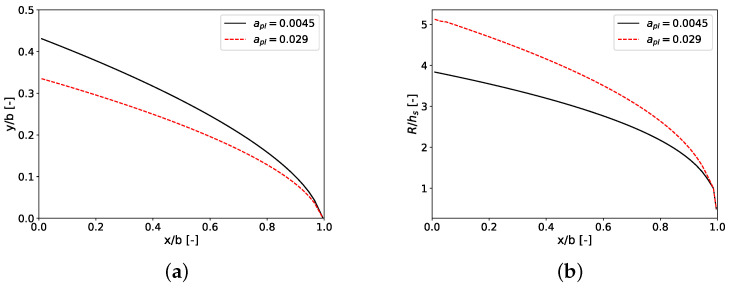
Design curves for different temperature sensitivity parameters (**a**) die land length *y*; (**b**) manifold radius; see Table 8 for values of the parameters used.

**Figure 12:**
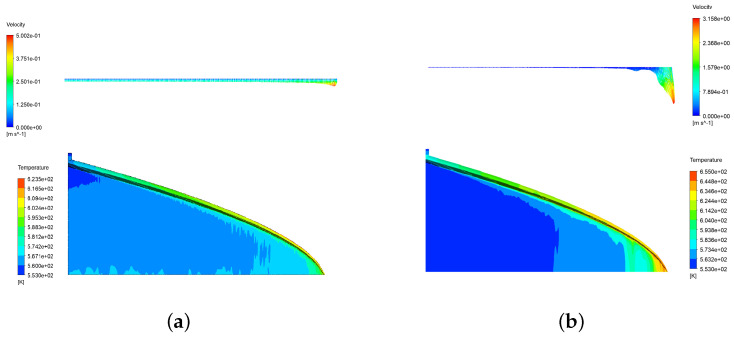
Velocity distribution at die exit and temperature distribution at the surface of the die for temperature sensitivity parameter (**a**) $\alpha_{pl}=0.0045$ K ${}^{-1}$ and (**b**) $\alpha_{pl}=0.029$ K ${}^{-1}$; see Table 8 for values of the parameters used.

Design curves for temperature sensitivity parameters $\alpha_{pl}$ of 0.0045 K ${}^{-1}$ and 0.029 K ${}^{-1}$ are shown in Figure 11. Other physical properties and process conditions are given in Table 8. Figure 12 shows exit flow distribution of nonisothermal design at the die exit for both temperature sensitivity parameters. The CFD-obtained velocity uniformity indices for $\alpha_{pl}$ of 0.0045 K ${}^{-1}$ and 0.029 K ${}^{-1}$ are 0.280732 and 1.31557, respectively. This shows the importance of this parameter on the effectiveness of the proposed model.

In Figure 12, contours depict temperature distribution on the interior wall of the die. Temperature increases in the manifold results in lower viscosity and lower resistance to fluid flow, and therefore a higher percentage of polymer melts tends to flow through the manifold with higher velocity at the die exit (see Figure 12b). For high values of temperature sensitivity parameters, a better control of temperature rather than simplified assumption of Biot of 1.0 is required. It can be concluded that the temperature boundary condition with a Biot number of a numerical value of 1.0 gives only adequate results for lower values of temperature sensitivity parameters.

## 4. Conclusions

The previously proposed modified Winter–Fritz constant shear rate isothermal design is improved to take into account temperature dependency and effect of viscous dissipation. The new design is compared with the isothermal design through 3D CFD simulation. The temperature increase due to the viscous dissipation and its effect on hydrodynamics of extrusion die is illustrated with numerical simulation results. A Brinkman number is defined to quantify the effect of viscous dissipation in the extrusion die performance. It is shown that, for low to medium Brinkman numbers, the proposed design results in better velocity distribution at the exit of the die compared to previous isothermal design at a higher impact region of the Brinkman numbers. For example, when the Brinkman number is equal to 365, the proposed design results in 5.24% improvement in a velocity uniformity level at the die exit in the example. Assumption of a unidirectional fully-developed velocity profile along the channel is discussed, and it is shown that, due to temperature increase and dependency of viscosity, a fully-developed velocity profile tends to flatten. In addition, the effect of the temperature sensitivity parameter is also studied, and it is proposed to employ an adaptive temperature boundary condition that negates the effect of an excessive temperature increase in the manifold.

## Figures and Tables

**Table 1:** Non-dimensionalization used in the energy equation.

Parameter	Dimension	Non-Dimensional
distance	$x_{1}$	${\tilde{x}}_{1}=x_{1}/L_{c}$
axial distance	$x_{2}$	${\tilde{x}}_{2}=x_{2}/\left(\mathrm{Pe}\;L_{c}\right)$
velocity	*u*	$\tilde{u}=u/\left({\dot{\gamma }}_{w}L_{c}\right)$
temperature	*T*	$\tilde{T}=\left(T-T_{R}\right)/\left(T_{0}-T_{R}\right)$

**Table 2:** Non-dimensionalized power-law viscosity function.

	$\tilde{\eta }\left(S,\tilde{T}\right)=a_{T}^{\prime }S^{n-1}$
$a_{T}$	$m_{0,pl}^{\prime }e^{-a_{pl}^{\prime }\tilde{T}}$
$m_{0,pl}^{\prime }$	$e^{-\alpha_{pl}\left(T_{R}-T_{ref}\right)}$
$\alpha_{pl}^{\prime }$	$\alpha_{pl}\left(T_{0}-T_{R}\right)$

**Table 3:** Non-dimensionalized Carreau–Yasuda viscosity function.

	$\tilde{\eta }\left(S,\tilde{T}\right)=a_{T}^{\prime }\;{\hat{\eta }}_{\infty }+a_{T}^{\prime }\left({\hat{\eta }}_{0}-{\hat{\eta }}_{\infty }\right){\left[1+{\left(a_{T}^{\prime }\lambda^{\prime }S\right)}^{a}\right]}^{\frac{n-1}{a}}$
${\hat{\eta }}_{\infty }$	$\eta_{\infty }/\eta_{w}$
${\tilde{\eta }}_{0}$	$\eta_{0}/\eta_{w}$
$\lambda^{\prime }$	$\lambda \dot{\gamma_{w}}$
$a_{T}^{\prime }$	$m_{0,cs}^{\prime }e^{\alpha^{\prime }\tilde{T}}$
$m_{0,cs}^{\prime }$	$e^{\alpha_{cs}\left(T_{R}-T_{ref}\right)}$
$\alpha_{cs}^{\prime }$	$\alpha_{cs}\left(T_{0}-T_{R}\right)$

**Table 4:** Input parameters and physical properties for the design and optimization shown in Figure 6.

Parameter	Value
Flow rate at entry of die, $Q_{0}$	$5\times 10^{-5}\;m^{3}\;\cdot \;s^{-1}$
Land height, $h_{s}$	1.5mm
Half width of die, *b*	360mm
Temperature at entry, $T_{0}$	473.15K
Temperature of heater, $T_{R}$	463.15K
Thermal conductivity, $k_{t}$	$0.16\;W\;\cdot \;m^{-1}\;\cdot \;K^{-1}$
Density, ρ	$1150\;\mathrm{kg}\;\cdot \;m^{-3}$
Specific heat, $c_{p}$	$1800\;J\;\cdot \;{\mathrm{kg}}^{-1}\;\cdot \;K^{-1}$

**Table 5:** Parameters of cases for comparison in the study of the effect of viscous dissipation.

	Values
Power-law model parameter, *n*	0.296
Power-law model parameter, $\alpha_{pl}$	0.0045 $K^{-1}$
Power-law model parameter, $T_{\mathrm{ref}}$	503.15 K
Flow rate ($Q_{0}$)	1.99 $m^{3}/h$
Inlet temperature ($T_{0}$)	553.15 K
Heater temperature ($T_{R}$)	543.15 K
Half die width (*b*)	360 mm
Land height $\left(h_{s}\right)$	1.5 mm
Thermal diffusivity ($\alpha_{T}$)	$1.57\times 10^{-7}$ $m^{2}/s$

**Table 6:** Comparison of uniformity index ϕ obtained by CFD of different designs for different Brinkman numbers.

Design	Case 1	Case 2	Case 3	Case 4
$m_{0}$ [$\mathrm{Pa}\;\cdot \;s^{n}$]	100	5000	10,000	15,000
${\mathrm{Br}}_{\mathrm{die}}$	2.42	122	243	365
ϕ of Isothermal design	0.269825	0.270569	0.285509	0.304196
ϕ of nonisothermal design (This work)	0.269825	0.278016	0.276205	0.288235

**Table 7:** Input parameters for comparison of the CFD, one-dimensional and analytical velocity profiles; results are shown in Figure 10.

	Values
Power-law model parameter, *n*	0.296
Power-law model parameter, $m_{0}$	$1.5\times 10^{4}$ $\mathrm{Pa}\;\cdot \;s^{n}$
Power-law model parameter, $\alpha_{pl}$	0.0045 $K^{-1}$
Power-law model parameter, $T_{\mathrm{ref}}$	503.15 K
Pressure drop (Δp)	40 MPa
Length of channel (*L*)	0.155 m
Inlet temperature ($T_{0}$)	553.15 K
Heater temperature ($T_{R}$)	543.15 K
Distance between two plates ($L_{c}$)	1.5 mm
Thermal diffusivity ($\alpha_{T}$)	$1.57\times 10^{-7}$ $m^{2}/s$
Thermal boundary condition	Bi=1

**Table 8:** Process conditions and physical properties for comparison of the temperature sensitivity parameter as shown in Figure 11 and Figure 12.

Quantity	Values
Power-law model parameter, *n*	0.296
Power-law model parameter, $m_{0}$	$1.5\times 10^{4}$ $\mathrm{Pa}\;\cdot \;s^{n}$
Power-law model parameter, $T_{\mathrm{ref}}$	503.15 K
Flow rate ($Q_{0}$)	1.99 $m^{3}/h$
Inlet temperature ($T_{0}$)	553.15 K
Heater temperature ($T_{R}$)	543.15 K
Half die width (*b*)	360 mm
Land height $\left(h_{s}\right)$	1.5 mm
Thermal diffusivity ($\alpha_{T}$)	$1.57\;\times 10^{-7}$ $m^{2}/s$

## Data Availability

Data will be available on request.

## Nomenclature

Bi	Biot number
Br	Brinkman number
$Br_{die}$	overall Brinkman number
*L*	length of a segment
$L_{c}$	characteristic length (i.e., radius or height)
*N*	number of segments
Pr	Prandtl number
*Q*	flow rate
*R*	radius of manifold
*S*	non-dimensional shear rate
*T*	temperature
$T_{0}$	temperature at entry
$T_{ref}$	reference temperature in power-law and Carreau–Yasuda models
Δζ	length of a manifold segment
$\hat{T}$	non-dimensional temperature
$\hat{u}$	non-dimensional velocity
$\hat{x_{1}}$	non-dimensional transverse coordinate
$\hat{x_{2}}$	non-dimensional axial coordinate
U	velocity vector
$a_{T}$	temperature dependence function of viscosity model
*b*	half width of die
$c_{p}$	heat capacity
*h*	heat transfer coefficient
$T_{R}$	temperature of heater
$h_{s}$	land height
$k_{l}$	thermal conductivity
$m_{0}$	power-law model consistency parameter
*n*	power-law index
*p*	pressure
*u*	velocity
$x_{1}$	transverse coordinate
$x_{2}$	axial coordinate
y(x)	distance from center of manifold to die exit
**Greek letters**	
$\alpha_{T}$	thermal diffusivity
$\alpha_{cs}$	Carreau–Yasuda model parameter
$\alpha_{pl}$	power-law or Carreau–Yasuda model parameter
$\dot{\gamma }$	shear rate
ϵ	convergence criterion
η	viscosity
$\eta_{0}$	Carreau–Yasuda model parameter
$\eta_{\infty }$	Carreau–Yasuda model parameter
$\hat{\eta }$	non-dimensional viscosity
λ	Carreau–Yasuda model parameter
μ	apparent viscosity
ϕ	flow uniformity index
ρ	density
τ	stress tensor
**Subscripts**	
0	at entry
ave	average
m	manifold
s	slit
w	evaluated at wall
